# Current Approaches to the Functional Gastrointestinal Disorders

**DOI:** 10.1155/2017/4957154

**Published:** 2017-01-02

**Authors:** Branka Filipović, Alastair Forbes, Bojan Tepeš

**Affiliations:** ^1^Clinical and Hospital Center “Bežanijska Kosa”, Medical Faculty, University of Belgrade, Autoput s/n, 4/2 Dr. Subotića Starijeg, 11000 Belgrade, Serbia; ^2^Norwich Medical School, University of East Anglia, Bob Champion Building, James Watson Road, Norwich Research Park, Norwich NR4 7UQ, UK; ^3^ABAKUS MEDICO d.o.o., Diagnostični Center Rogaška, Prvomajska 29, SI-3250 Rogaška Slatina, Slovenia

Functional gastrointestinal disorders (FGD), namely, functional gastrointestinal disorders (FGID) and irritable bowel syndrome (IBS), are always in the focus of gastroenterologists, because of their polymorphic symptomatology and insufficiently defined causes and habits that influence the appearance of FGD. Irritable bowel syndrome (IBS) is a functional gastrointestinal disorder (FGID) characterized by abdominal pain or discomfort and alteration of bowel habits in the absence of an organic disorder. IBS is the most common gastrointestinal disorder and the prevalence varies from 4% to 22% in the population based studies [1–3]. Functional dyspepsia (FD) is common and significantly impairs quality of life. Symptoms of FD are considered to originate from the gastroduodenal region, classified by the Rome criteria as disorders of brain-gut interaction without structural alteration. However, it is now apparent that FD is a number of syndromes, the epigastric pain syndrome (bothersome epigastric pain or epigastric burning) and the postprandial distress syndrome (with bothersome postprandial fullness or early satiation) and there are wide-ranging symptoms and severity [4].

Papers in this special issue additionally illustrate the approach to FGD from various points of view. W.-P. Meng and coworkers presented a review article that describes the mutual effects of* H. pylori* and hormones in functional dyspepsia and provided new insight into the pathogenesis of functional dyspepsia.* H. pylori* strains have been shown to affect the secretion of several hormones, including 5-HT, ghrelin, dopamine, and gastrin, and altered levels of these hormones might be the cause of the psychological disorders of functional dyspepsia patients.

P. Enck and his team of investigators revealed that somatic comorbid condition and/or regular medication were significantly older than those with functional constipation and had lower health and social status, but similar general life satisfaction. Their quality-of-life was lower for the physical but not for the mental health domain, while among those with functional constipation, the mental health domain distinguished IBS-Constipation dominant type individuals from those with functional constipation but without pain.

C.-H. Yi and associates reported rectal decompression with either rectal or sham tube improved distension-induced abdominal symptoms. Their study indicates that the mechanisms that improved abdominal symptoms by rectal decompression might be mediated by a central pathway instead of a peripheral mechanism.

A well-designed study by W. Tao et al. identified a set of 35 differentially expressed miRNAs that bear the potential to be molecular markers of T2DM with D-IBS, as they clearly discriminated T2DM with D-IBS from healthy subjects. Furthermore, our findings identified three miRNA species, hsa-miR-106b, hsa-miR-26a, and hsa-miR-29b, which are differentially expressed in the peripheral circulation of patients suffering from T2DM with D-IBS and are among the miRNAs that are implicated in the MAKP signaling pathway.

Study of Chinese cohort with FD, provided by Chen et al., demonstrated that sleep quality and marriage status and introversion-extroversion of personality traits significantly influenced the conventional treatment effects on Chinese FD patients.

S. Jizhong and his colleagues outlined that IBS patients during stress released corticotrophin release factor which leads to the damage of the intestinal mucosa barrier. TLR2 and TLR4 activation produces an inflammatory reaction, which can concurrently affect the digestive tract and the CNS and induces the corresponding digestive and psychiatric symptoms.

All the investigations represent a contribution to clarify the pathogenesis of FGD, but, judging from the results presented, functional gastrointestinal disorder will attract the intention of investigators for a longer period, because the definitive solution about the pathogenesis of FGD and, consecutively, the definitive guidelines for the therapy remain yet to be elucidated.

*Branka Filipović* *Branka Filipović* *Alastair Forbes* *Alastair Forbes* *Bojan Tepeš* *Bojan Tepeš*
